# Platelet to lymphocyte and neutrophil to lymphocyte ratio in the first trimester of pregnancy, are they useful for predicting spontaneous miscarriage? A case-control study

**DOI:** 10.18502/ijrm.v21i6.13632

**Published:** 2023-07-24

**Authors:** Maryam Yazdizadeh, Nafisseh Hivehchi, Marjan Ghaemi, Sepideh Azizi, Maryam Saeedzarandi, Narjes Afrooz, Pegah Keshavarz, Melika Ansarin, Maliheh Fakehi, Mina Yazdizadeh, Mojgan Mokhtari

**Affiliations:** ^1^Shahid Akbarabadi Clinical Research Development Unit, School of Medicine, Iran University of Medical Sciences, Tehran, Iran.; ^2^Vali-E-Asr Reproductive Health Research Center, Family Health Research Institute, Tehran University of Medical Sciences, Tehran, Iran.; ^3^Oral Health Research Center, Health Research Institute, Babol University of Medical Sciences, Babol, Iran.

**Keywords:** Spontaneous abortion, Inflammation, Neutrophils, Lymphocytes, Blood platelet, Pregnancy.

## Abstract

**Background:**

In 15% of all clinical pregnancies, a miscarriage can occur, but the exact cause of this phenomenon is not fully understood. However, it is believed that a faulty placenta, which triggers an inflammatory response in the mother's body, may be one of the causes. Medical literature has increasingly focused on 2 indicators of inflammation, the platelet-lymphocyte ratio (PLR) and neutrophil-lymphocyte ratio (NLR). Despite this, there has yet to be a study conducted that examines the rates of PLR and NLR in cases of miscarriage.

**Objective:**

This study aims to determine whether there is an increase in complete blood count inflammatory parameters such as NLR and PLR in women who experience miscarriages.

**Materials and Methods:**

This retrospective case-control study was conducted from March 2021 to March 2022, across 3 academic hospitals in Tehran, Iran. A total of 240 participants were enrolled comprising individuals with either miscarriages or normal pregnancies (n = 120/each). Data were collected from the medical records of participants aged between 18-42 yr old, with gestational age ranging from 6-13 wk. The demographic information, including age, body mass index, parity, history of abortion, number of abortions, number of living children, hematocrit and hemoglobin levels, platelet distribution width (PDW), PLR, NLR, mean platelet volume, and platelet were extracted from their records. The gestational age was also recorded.

**Results:**

A total of 240 participants were recruited for the study. PDW, NLR, PLR, and lymphocyte values were higher in the miscarriage group compared to the healthy normal pregnant women (p < 0.001). Mean platelet volumes were found to be lower in the miscarriage group compared to the healthy normal pregnant women (p < 0.001).

**Conclusion:**

Although, no statistically significant difference was observed in the hemoglobin, hematocrit, platelets, and neutrophils in these 2 groups of pregnant women. The higher inflammatory markers including PDW, NLR, and PLR could potentially aid in the speculation of defective placentation as a contributing factor to the development of miscarriage. Measurement of these markers may be useful to predict pregnancy leading to miscarriage.

## 1. Introduction

Miscarriage is defined as an intrauterine death of the embryo or fetus without expulsion of conception products, which takes place roughly in 15% of all clinically recognized pregnancies (1). It is usually an appalling incident that may lead to numerous maternal complications such as anemia, injuries to the uterus and endometrium, and infection (2).

Placental-related diseases, such as preeclampsia or missed abortion, are strongly impacted by oxidative stress or an imbalance in oxidant/antioxidant activity in the tissues of the uteroplacental region. During the initial stages of development, the human fetus is exposed to a low oxygen environment. This natural hypoxia in the first trimester may serve as a protective mechanism against the harmful and teratogenic effects of oxygen free radicals. A delicate equilibrium between the metabolic requirements of the fetus and its placenta, as well as the potential risks posed by free radicals, is crucial for the healthy progression of a pregnancy. Any factor inducing an unusual concentration of O ${}_{2}$ would lead to O ${}_{2}$ -mediated damage and increased apoptosis and necrosis of the trophoblastic epithelium (3).

Hence, the immune response alters resulting in abnormal placental function, with syncytiotrophoblast ischemia and the appearance of products that are the critical reasons for endothelial integrity damage. The occurrence of endothelial dysfunction triggers an inflammatory response within blood vessels that involves intravascular leukocytes, as well as the clotting and complement systems (4).

It is believed that the elevation in systemic inflammatory response markers including a neutrophil-lymphocyte ratio (NLR), platelet-lymphocyte ratio (PLR), red cell distribution width, mean platelet volume (MPV), and plateletcrit are related to the pathology of preeclampsia (5). Regarding the similar inflammatory cascade in preeclampsia and missed abortion pathophysiology, we hypothesize that these markers would have notable alterations in miscarriage.

Complete blood count (CBC), a simple, inexpensive, practical, and highly sensitive test, is one of the routine screening tests during pregnancy. All the above-mentioned markers can be extracted from the CBC test results. Considering the paramount importance of finding out the diagnostic and prognostic factors associated with miscarriage, the results of this study will be of much help in minimizing the occurrence of miscarriage.

In the literature, several studies have focused on NLR and PLR in pathologic conditions such as gynecological cancers (6), gestational diabetes (7), and endometriosis (3). Also, the power of predicting early pregnancy loss and missed miscarriage of NLR, PLR, and MPV values were studied. In a case-control study, NLR was known to be a positive predictive marker, while PLR and MPV were found to be negative predictive markers for spontaneous abortion (8). However, in another study, NLR and PLR values were found to be without any determining influence on the occurrence of missed miscarriage (9). In addition, in a retrospective study of cases with miscarriages, they found out that NLR is the only prognostic value in early miscarriages (10).

This study aims to investigate the NLR and PLR values in miscarriage and normal pregnancies.

## 2. Materials and Methods

This retrospective case-control study was conducted from March 2021 to March 2022 in 3 referral academic hospitals in Tehran, Iran. A total of 240 participants were divided into 2 groups (n = 120/ each group) including miscarriage and the normal pregnancy groups. A structured data-extracting (checklist) tool was developed and pretested before the actual data collection. Data were collected from the records of participants aged between 18 and 42 yr old with 6-13 wk of gestation. The miscarriage was defined by the absence of detectable embryonic cardiac activity in ultrasound at least 24 hr apart, whereas detecting normal fetal heart rate and activity in 2 distinct ultrasounds was recognized as normal pregnancies. If no fetal cardiac activity was observed in the first ultrasound, then it was re-performed. If the fetal heart rate was still undetectable, it was considered a miscarriage. However, if any signs of fetal cardiac activity were detected, the ultrasound was performed again to confirm the fetal heart rate in at least 2 distinct ultrasounds.

The study group was selected from the cases with normal pregnancies and miscarriages according to the above-mentioned definitions. The exclusion criteria were thyroid disorders, diabetes mellitus, hematologic disease, history of thrombosis, systemic lupus erythematosus, or multiple gestations, smoking, drug or alcohol abuse, ongoing infection, malignancies, chronic inflammatory disease, participants who were under certain medications like anti-inflammatory agents or corticosteroids, all pregnant women who experienced miscarriage notwithstanding having a normal pregnancy at the time of the study, a diagnosed pathology claimed to be contributing to abortion, such as uterine abnormalities, body mass index (BMI) > 25 kg/m^2^, anembryonic pregnancy.

The gestational age was calculated as the beginning of the first day of the last menstrual period, which was established regarding the crown-rump length values in ultrasound in 8-14 wk of gestation. Other demographic data, including age, BMI, parity, history of abortion, number of abortions, number of alive children, hematocrit and hemoglobin levels, platelet distribution width (PDW), PLR, NLR, MPV, and platelet were extracted from their records.

### Ethical considerations 

The study was approved by the Iran University of Medical Sciences, Tehran, Iran and the Medical Ethics Committee (Code: IR.IUMS.FMD.REC.1400.311).

### Statistical analysis

Data analysis was performed by IBM Statistical Package for the Social Sciences, version 22.0, SPSS Inc., Chicago, Illinois, USA (SPSS) for windows. Variables were listed as mean ± standard deviation. An independent sample *t* test and Mann-Whitney U test was performed to compare the missed abortion group and the normal pregnancy group in all continuous variables. Logistic regression was used to identify predicting values of missed abortion, and receiver operating characteristic (ROC) was used to investigate the diagnostic performance of NLR and PLR values between the case and the control group. The odds ratio and 95% confidence interval were used to estimate the associations between the underlying factors and the missed abortion. Statistical significance was defined as p < 0.05.

## 3. Results

Of the 240 participants included in this study, 120 were diagnosed with miscarriage, whereas 120 were experiencing a normal pregnancy, thus considered as the control group. After confirming the eligibility of the cases to be included in our study, the required data were extracted from the medical record meticulously. The data on participant demographics are summarized in table I, along with comparative statistics which showed no significant between-group differences for age, weight, height, BMI, and gestational age.

Table II shows the obstetrics characteristics between groups. The gravidity, parity, and current living children were roughly similar between the case and control groups. Of note, there were significantly more abortions in the history of participants with currently missed abortions (p < 0.001).

Table III shows hematologic indices of pregnant women with either miscarriages or normal pregnancies. The results show no significant differences were observed in hemoglobin, hematocrit, platelet, and neutrophil levels between the case and the control group (p > 0.05). However, MPV and PDW levels were relatively higher in women with miscarriages compared to normal pregnancies (p < 0.001, p = 0.001). The mean of lymphocytes in women with miscarriage was 1.77 and in normal pregnancies was 2.29, which were statistically different (p < 0.001).

The comparison of NLR and PLR showed that these values were significantly higher in women with miscarriages compared to the control group (p < 0.001). According to the area under the ROC Curve, NLR is a relatively more sensitive value for clinical settings.

**Figure 1:**
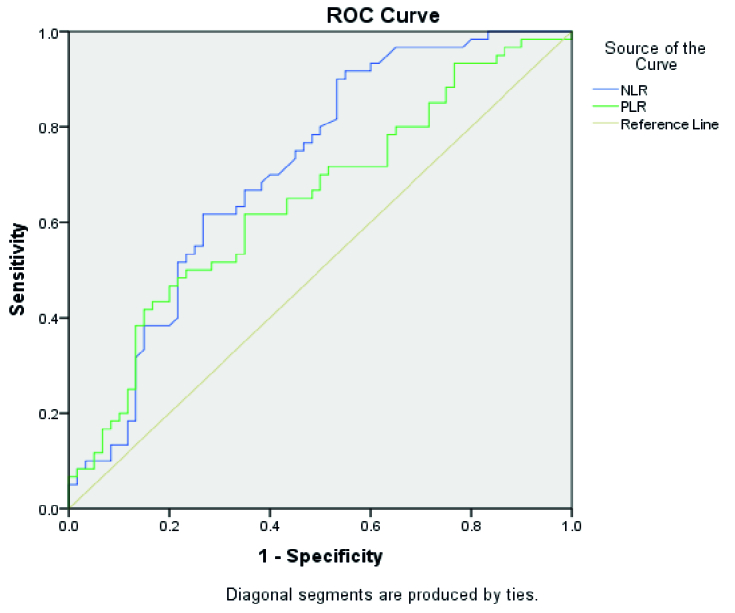
Receiver operating characteristic curve to determine the diagnostic performance of neutrophil to lymphocyte ratio (NLR) and platelet to lymphocyte ratio (PLR).

Figure 1 shows the ROC curve to determine the diagnostic performance of NLR and PLR. In table IV, the results of the ROC curve analysis are shown. According to the area under the curve, which is reported as 71.7% for NLR and 64.6% for PLR, the diagnostic value of NLR was better in the miscarriage cases.

Table V shows the logistic regression model that was used to estimate the relationship between the potential factors and miscarriages. The results show that the incidence of miscarriage in women with previous miscarriages was 8.820 times higher than in women without prior history of abortion (p < 0.001). Raised levels of MPV reduced the risk of miscarriages (p < 0.001). The possibility of missed abortion grew when PDW was lower. A rise in each unit of PDW was associated with 1.903 times higher risk of missed abortion (p < 0.001).

Furthermore, it appears that there is a positive correlation between the rise in NLR and the miscarriage risk. An increase in each unit of NLR was associated with a 1.453 times more elevated possibility of miscarriage (p = 0.038).

**Table 1:** Patient characteristics in 2 groups consisted of miscarriage and normal pregnancy


**Variables**	**Total**	**Miscarriage**	**Normal pregnancy**	**P-value**
**Age (yr)**	30.29 ± 4.31	30.46 ± 4.44	30.13 ± 4.18	0.55
**Weight (kg)**	61.68 ± 5.54	61.73 ± 5.44	60.63 ± 5.66	0.88
**Height (cm)**	166.20 ± 5.86	165.62 ± 6.09	166.78 ± 5.59	0.12
**BMI (kg/m^2^)**	22.34 ± 1.75	22.52 ± 1.77	22.16 ± 1.73	0.10
**Gestational age (days)**	59.63 ± 5.36	59.98 ± 4.85	59.28 ± 5.82	0.31
Data presented as Mean ± SD. Independent *t* test. BMI: Body mass index				

**Table 2:** Obstetrics characteristics of participants in 2 groups consisted of missed abortion and normal pregnancy


**Variables**	**Total**	**Miscarriage**	**Normal pregnancy**	**P-value**
**Gravida**	2.40 ± 0.91 (2)	2.42 ± 1.10 (2)	2.38 ± 0.66 (2)	0.80
**Parity**	1.04 ± 0.46 (1)	1.03 ± 0.45 (1)	1.05 ± 0.47 (1)	0.77
**Previous abortion**	0.86 ± 0.93 (1)	1.38 ± 0.95 (1)	0.33 ± 0.51 (1)	< 0.001
**Living children**	1.04 ± 0.46 (1)	1.03 ± 0.45 (1)	1.05 ± 0.47 (1)	0.77
Data presented as Mean ± SD (median). Mann-Whitney U test				

**Table 3:** Hematologic indices in 2 groups consisting of missed abortion and normal pregnancy


**Variables**	**Total **	**Miscarriage**	**Normal pregnancy**	**P-value**
**Hemoglobin**	12.63 ± 1.02	12.74 ± 1.03	12.52 ± 1.00	0.09
**Hematocrit**	38.77 ± 4.75	39.15 ± 4.73	38.38 ± 4.75	0.20
**PTL**	258.01 ± 72.62	258.66 ± 78.76	257.36 ± 66.24	0.89
**MPV**	9.76 ± 9.60	9.42 ± 1.52	10.11 ± 1.12	< 0.001
**PDW**	16.58 ± 1.14	16.81 ± 1.26	16.34 ± 0.96	< 0.001
**Neutrophil**	5.45 ± 1.56	5.58 ± 1.45	5.32 ± 1.66	0.20
**Lymphocyte**	2.03 ± 0.77	1.77 ± 0.61	2.29 ± 0.83	< 0.001
**NLR**	3.07 ± 1.50	3.51 ± 1.53	2.62 ± 1.33	< 0.001
**PLR**	149.03 ± 76.42	168.67 ± 84.41	129.39 ± 61.84	< 0.001
Data presented as Mean ± SD. Independent *t* test. PTL: Platelet, MPV: Mean platelet volume, PDW: Platelet distribution width, NLR: Neutrophil-lymphocyte ratio, PLR: Platelet-lymphocyte ratio				

**Table 4:** Results of receiver operating characteristic curve analysis


		**95% Confidence Interval**	
**Variables**	**Area under curve**	**Standard deviation**	**P-value**	**Lower limit**	**Upper limit**
				**NLR**	0.71	0.03	< 0.001	0.64	0.77
**PLR**	0.64	0.03	< 0.001	0.57	0.71
NLR: Neutrophil-lymphocyte ratio, PLR: Platelet-lymphocyte ratio					

**Table 5:** Prognostic factors of miscarriage based on logistic regression analysis


	**B**	**OR (95% CI)**	P-value*
**Variables**	-9.200	—	0.02
	**Previous miscarriage**	2.177	8.82 (4.82-16.11)	< 0.001
**Hemoglobin**	0.116	1.12 (0.80-1.57)	0.50
**MPV**	-0.532	0.58 (0.43-0.78)	< 0.001
**PDW**	0.643	1.90 (1.31-2.76)	< 0.001
**Lymphocyte**	-0.311	0.73 (0.31-1.70)	0.47
**NLR**	0.347	1.45 (1.02-2.06)	0.03
**PLR**	0.000	1.00 (0.99-1.00)	0.92
*Logistic regression. MPV: Mean platelet volume, PDW: Platelet distribution width, NLR: Neutrophil-lymphocyte ratio, PLR: Platelet-lymphocyte ratio			

## 4. Discussion

This study showed a significant increase in PDW, lymphocytes, NLR, and PLR in miscarriage and a decrease in MPV. In addition, our study shows that the ability of NLR to predict miscarriage (as measured by area under the curve) is superior to that of PLR.

As mentioned, the defective placentation and reduced cytotrophoblast invasion of the endometrium, and partial plugging of the lumen of the spiral arteries are the anatomical clarification of the pathophysiology of many early pregnancy failures. In case of increased O_2_ and lipid peroxidase concentration, placental degeneration, dysfunction of syncytiotrophoblast, and detachment of the placenta from the uterine wall would be inevitable.

As preeclampsia and miscarriage are both placental-related diseases, and preeclampsia stems from a similar defect in trophoblast invasion, the correlation of inflammatory markers and preeclampsia might be detected in miscarriage as well. It is now believed that dysregulated immune cell response and consequent immunologic abnormality plays a crucial role in pregnancy loss. It has been stated that in both early pregnancy failures and preeclampsia, the inflammation markers and oxidative biomarkers are elevated in maternal blood significantly (11). The elevation of markers is investigated in preeclampsia, but to date, there are limited studies investigating them in miscarriage.

In this study, we found that the baseline PDW, NLR, and PLR tend to be significantly higher in missed abortions compared to normal pregnancies. However, MPV levels were found to be lower in missed abortions compared to the control group. These findings are consistent with the results of another study, which concluded that NLR and PLR are higher in miscarriage compared to normal pregnancies (12). Also, we contend that the assumption that the NLR and PLR have equal diagnostic value for missed abortion is likely to be incorrect.

It is demonstrated that NLR and PLR are significantly higher in early abortion cases compared to the control group, which included women with term deliveries (13). On the other hand, in a recent study, it is stated that NLR and PLR are lower in inevitable abortion compared to induced abortion and normal pregnancies (14).

It is reported that the risk of spontaneous abortion tended to be higher as NLR increased, while PLR was a negative diagnostic factor in evaluating spontaneous abortion. Nevertheless, in another study PLR was relatively higher in patients with abortion and NLR was not significantly different from the control group (8). Surprisingly, Gorkem and colleagues found that there are no meaningful differences among normal pregnancy, induced abortion, and spontaneous abortion cases in terms of PLR and NLR. The results of this study were not consistent with our results (15). Taking into consideration the discrepancies in the results, we assumed that these differences were possibly the result of using different methods or equipment and the presence or absence of confounder factors among the included participants.

In general, women with miscarriages were likely to have higher NLR, PLR, and PDW but lower MPV in comparison to the control group. There has been no available data on the rise of MPV in miscarriage until now. It is shown that MPV is similar between normal pregnancies and women with abortions. It is shown that PLR and MPV are lower in spontaneous abortion and NLR, lymphocyte, and neutrophil are higher in that group (16).

Also, we believe that the detected increase in PDW in missed abortion cases may be an indicator of endothelial and placental injury, resulting in a higher probability of thrombosis. Based on the literature, there are strong evidence that the cytokine levels like tumor necrosis factor alpha, interferon-gamma, interleukin (IL)-2, IL-6, IL-10, and IL-12 are elevated in abortions (11, 17, 18). However, having access to CBC at minimal cost and inconvenience and being taken in all pregnant women as a part of screening tests, makes it a more appealing way to detect or predict abnormalities.

The results from the present study may provide helpful references for a deeper understanding of missed abortion pathophysiology and more convenient ways to predict it. Since NLR could be quickly calculated based on a routine screening test, clinicians may identify high-risk pregnant women at an early stage. Thus, special care can be provided to reduce the occurrence of abortions.

The sample size was the limitation of this study. It is recommended that in future studies more participants from various ethical and racial backgrounds should be included. Moreover, we did not evaluate the NLR, PLR, and other hematologic indices before pregnancy. The comparison of the values before, during, and after the pregnancy should be a priority in upcoming studies.

## 5. Conclusion

In conclusion, regarding the higher levels of PLR, NLR, and PDW in women with missed abortion, this study confirms that the elevation in NLR and PLR may be an indicator of injury to the trophoblast and placental defect. There is a significant correlation between the level of the mentioned values and missed pregnancy.

##  Conflict of Interest

The authors declare that there is no conflict of interest.

## References

[B1] Wu H-L, Marwah S, Wang P, Wang Q-M, Chen X-W (2017). Misoprostol for medical treatment of missed abortion: A systematic review and network meta-analysis. Sci Rep.

[B2] Cunningham F, Leveno KJ, Bloom SL, Dashe JS, Hoffman BL, Casey B, et al

[B3] Yang X, Hu R, Shi M, Wang L, Yan J, Gong J, et al (2023). Placental malfunction, fetal survival and development caused by sow metabolic disorder: The impact of maternal oxidative stress. Antioxidants.

[B4] Mihu D, Razvan C, Malutan A, Mihaela C (2015). Evaluation of maternal systemic inflammatory response in preeclampsia. Taiwan J Obstet Gynecol.

[B5] Yücel B, Ustun B (2017). Neutrophil to lymphocyte ratio, platelet to lymphocyte ratio, mean platelet volume, red cell distribution width and plateletcrit in preeclampsia. Pregnancy Hypertens.

[B6] Ethier J-L, Desautels DN, Templeton AJ, Oza A, Amir E, Lheureux S (2017). Is the neutrophil-to-lymphocyte ratio prognostic of survival outcomes in gynecologic cancers? A systematic review and meta-analysis. Gynecol Oncol.

[B7] Sargın MA, Yassa M, Taymur BD, Celik A, Ergun E, Tug N

[B8] Bas FY, Tola EN, Sak S, Cankaya BA (2018). The role of complete blood inflammation markers in the prediction of spontaneous abortion. Pak J Med Sci.

[B9] Liu D, Huang X, Xu Z, Chen M, Wu M (2022). Predictive value of NLR and PLR in missed miscarriage. J Clin Lab Anal.

[B10] Kim Y (2020). Retrospective analysis of prognostic value of the neutrophil-to-lymphocyte ratio in early miscarriages: A 8-year survey. Medicine.

[B11] Lee ED, Mistry HD (2022). Placental related disorders of pregnancy. Int J Mol Sci.

[B12] Biyik I, Albayrak M, Keskin F (2020). Platelet to lymphocyte ratio and neutrophil to lymphocyte ratio in missed abortion. Rev Bras Ginecol Obstet.

[B13] Oğlak SC, Aydın MF

[B14] Yakıştıran B, Tanacan A, Altınboğa O, Yücel A

[B15] Gorkem U, Kan O, Bostanci MO, Taskiran D, Inal HA (2021). Kisspeptin and hematologic parameters as predictive biomarkers for first-trimester abortions. Medeni Med J.

[B16] Kosus N, Kosus A, Yildirim M, Duran M, Turhan NO (2011). Mean platelet volume as a marker of thrombosis in patients with missed abortion. Acta Haematol.

[B17] Chavan AR, Griffith OW, Wagner GP (2017). The inflammation paradox in the evolution of mammalian pregnancy: Turning a foe into a friend. Curr Opin Genet Dev.

[B18] Vilotić A, Nacka-Aleksić M, Pirković A, Bojić-Trbojević Z, Dekanski D, Krivokuća MJ (2022). IL-6 and IL-8: An overview of their roles in healthy and pathological pregnancies. Int J Mol Sci.

